# Improving and Predicting Outcomes of Traumatic Brain Injury: Neuroplasticity, Imaging Modalities, and Perspective Therapy

**DOI:** 10.1155/2017/4752546

**Published:** 2017-06-22

**Authors:** Chih-Lung Lin, Aaron S. Dumont, John H. Zhang, Mario Zuccarello, Cheng-Sheng Chen

**Affiliations:** ^1^Graduate Institute of Medicine, College of Medicine, Kaohsiung Medical University, Kaohsiung, Taiwan; ^2^Department of Neurosurgery, Kaohsiung Medical University Hospital, Kaohsiung, Taiwan; ^3^Department of Neurosurgery, Tulane University, New Orleans, LA 70112, USA; ^4^Departments of Neurosurgery, Physiology, and Anesthesiology, Loma Linda University, School of Medicine, Loma Linda, CA 92354, USA; ^5^Department of Neurosurgery, University of Cincinnati, Cincinnati, OH 45219, USA; ^6^Department of Psychiatry, Kaohsiung Medical University Hospital, Kaohsiung, Taiwan

Each year, a new traumatic brain injury (TBI) event occurs in an estimated 10 million people worldwide, particularly in young adults. Not only is TBI the leading cause of long-term disability and mortality worldwide, but it is also expected to become the third largest cause of global disease burden by 2020 [1, 2]. TBI is a challenging disease process, both to treat and to investigate. TBI survivors often experience substantial and lifelong cognitive, physical, and behavioral impairments that require long-term access to health care and disability services.

Over the past three decades, imaging modalities, such as positron emission tomography (PET), functional MRI (fMRI), diffusion tensor imaging (DTI), and transcranial magnetic stimulation (TMS), have played a pivotal role in predicting TBI outcome and advancing TBI treatment [3].

Burgeoning evidences for neuroplasticity have shed light on the potential therapeutic protocols focusing on synaptic proteins, new network connections, inflammatory reactions, and the recruitment of immune cells [4]. Future therapies, including gene therapies or a combination of different pharmacologic therapies and rehabilitative protocols, which may benefit victims by targeting multiple mechanisms of recovery, are of utmost interest and currently under heavy investigation by devoting neuroscientists.

The articles contained in this special issue include 4 reviews and 2 original research papers: a quantitative study focusing on predictors of recovery from TBI and a cohort study on substance related disorder after TBI.

- TBI survivors suffer various functional and cognitive sequelae that may impose serious medical and social problems. J. Ma et al. reviewed the complicated pathological mechanisms of diffuse axonal injury (DAI) in an attempt to facilitate more accurate diagnosis and hence improve the survival and life quality of DAI patients.
- Not many studies have discussed the role of synapses after TBI. Z. Wen et al. provided a comprehensive review on the role and mechanisms of synapses in TBI and the correlation between key synaptic proteins and neuroplasticity. The article also provides insights on the role of synapses in the treatment and prognosis of TBI.
- Molecular studies concerning the microglia-induced inflammation by M1 phenotype and anti-inflammation by M2 phenotype are a new strategy for treatment of TBI. In the paper titled “The Polarization States of Microglia in TBI: A New Paradigm for Pharmacological Intervention,” H. Xu et al. examined research on the polarization of microglia and their roles in the inflammation response and secondary brain injury after TBI. It is hoped that decreasing M1 phenotype and increasing M2 phenotype may shed light on the pharmacotherapy of TBI.
- Studies to locate the clinical predictors of recovery from prolonged disorders of consciousness (PDC) can be arduous. In the original research presented by H. Abe et al., 14 TBI patients were investigated using diffusion tensor imaging (DTI) for long-term follow-ups of 1-2 years. The results disclosed correlation between initial severity of PDC and difference in axial diffusivity (AD) and the degree of recovery from PDC (RPDC). Microstructural white matter changes in this study implicate their possible relationship with the degree of RPDC.
- Efforts to correct behavioral, cognitive, mood, and executive impairment of TBI patients are costly. The article “Rehabilitation Treatment and Progress of Traumatic Brain Injury Dysfunction” by B. Dang et al. compiles the current rehabilitation treatment plans and outcomes of TBI in adults.
- Whether TBI induces substance-related disorder (SRD) is currently debatable. C.-H. Wu et al. carried out a cohort study of 19 thousand TBI adults with no history of mental disorders prior to brain injury in the original paper titled “Traumatic Brain Injury and Substance Related Disorder: A 10-Year Nationwide Cohort Study in Taiwan.” Results show that the overall incidence of SRD was 3.62-fold higher in the TBI group and 9.01-fold in the severe TBI group. The severity of TBI seems to have strong correlation in the subsequent risks of SRD.

## References

[1] Hyder A. A., Wunderlich C. A., Puvanachandra P., Gururaj G., Kobusingye O. C. (2007). The impact of traumatic brain injuries: a global perspective. *Neuro Rehabilitation*.

[2] Shi H. Y., Hwang S. L., Lee I. C., Chen I. T., Lee K. T., Lin C. L. (2014). Trends and outcome predictors after traumatic brain injury surgery: a nationwide population-based study in Taiwan. *Journal of Neurosurgery*.

[3] Eierud C., Craddock R. C., Fletcher S. (2014). Neuroimaging after mild traumatic brain injury: review and meta-analysis. *Neuroimage Clinical*.

[4] Werner J. K., Stevens R. D. (2015). Traumatic brain injury: recent advances in plasticity and regeneration. *Current Opinion in Neurology*.

